# The Oxford Agoraphobic Avoidance Scale

**DOI:** 10.1017/S0033291721002713

**Published:** 2023-03

**Authors:** Sinead Lambe, Jessica C. Bird, Bao Sheng Loe, Laina Rosebrock, Thomas Kabir, Ariane Petit, Sophie Mulhall, Lucy Jenner, Charlotte Aynsworth, Elizabeth Murphy, Julia Jones, Rosie Powling, Kate Chapman, Robert Dudley, Anthony Morrison, Eileen O. Regan, Ly-Mee Yu, David Clark, Felicity Waite, Daniel Freeman

**Affiliations:** 1 Department of Psychiatry, University of Oxford, Oxford, UK; 2 Oxford Health NHS Foundation Trust, Oxford, UK; 3 NIHR Oxford Health Biomedical Research Centre, Oxford, UK; 4 The Psychometrics Centre, University of Cambridge, Cambridge, UK; 5 The McPin Foundation, London, UK; 6 Cumbria, Northumberland, Tyne and Wear NHS Foundation Trust, Newcastle upon Tyne, UK; 7 Greater Manchester Mental Health NHS Foundation Trust, Manchester, UK; 8 Nottinghamshire Healthcare NHS Foundation Trust, Nottingham, UK; 9 Avon and Wiltshire Mental Health Partnership NHS Trust, Bath, UK; 10 Newcastle University, Newcastle upon Tyne, UK; 11 Division of Psychology and Mental Health, University of Manchester, Manchester, UK; 12 Primary Care Clinical Trials Unit, Nuffield Department of Primary care Health Sciences, University of Oxford, Oxford, UK; 13 Department of Experimental Psychology, University of Oxford, Oxford, UK

**Keywords:** Assessment, psychosis, agoraphobic avoidance, social withdrawal

## Abstract

**Background:**

Agoraphobic avoidance of everyday situations is a common feature in many mental health disorders. Avoidance can be due to a variety of fears, including concerns about negative social evaluation, panicking, and harm from others. The result is inactivity and isolation. Behavioural avoidance tasks (BATs) provide an objective assessment of avoidance and *in situ* anxiety but are challenging to administer and lack standardisation. Our aim was to draw on the principles of BATs to develop a self-report measure of agoraphobia symptoms.

**Method:**

The scale was developed with 194 patients with agoraphobia in the context of psychosis, 427 individuals in the general population with high levels of agoraphobia, and 1094 individuals with low levels of agoraphobia. Factor analysis, item response theory, and receiver operating characteristic analyses were used. Validity was assessed against a BAT, actigraphy data, and an existing agoraphobia measure. Test–retest reliability was assessed with 264 participants.

**Results:**

An eight-item questionnaire with avoidance and distress response scales was developed. The avoidance and distress scales each had an excellent model fit and reliably assessed agoraphobic symptoms across the severity spectrum. All items were highly discriminative (avoidance: *a* = 1.24–5.43; distress: *a* = 1.60–5.48), indicating that small increases in agoraphobic symptoms led to a high probability of item endorsement. The scale demonstrated good internal reliability, test–retest reliability, and validity.

**Conclusions:**

The Oxford Agoraphobic Avoidance Scale has excellent psychometric properties. Clinical cut-offs and score ranges are provided. This precise assessment tool may help focus attention on the clinically important problem of agoraphobic avoidance.

## Introduction

For many people with mental health problems it can be a challenge to step out of the front door. Everyday activities – catching a bus, shopping locally, walking down the street – are avoided. This type of withdrawal from everyday life is a common feature of depression (Kennedy, Foy, Sherazi, McDonough, & McKeon, 2007), psychosis (Freeman, Taylor, Molodynski, & Waite, 2019a), anxiety disorders (Saris, Aghajani, van der Werff, van der Wee, & Penninx, 2017), and post-traumatic stress disorder (PTSD; Zayfert, DeViva, and Hofmann, 2005). Withdrawal often persists even after the mental health problem has remitted (Davidson, Dowrick, & Gunn, 2016; Kennedy et al., 2007). Physical and mental health are negatively affected by withdrawal (Patterson & Veenstra, 2010). Our view is that this withdrawal is – at least in part – driven by agoraphobic anxious avoidance (Freeman et al., 2019a, 2019b; McKnight, Monfort, Kashdan, Blalock, & Calton, 2016). Agoraphobia is characterised by fear and avoidance of places or situations where escape may not be possible or help not available (American Psychiatric Association, 2013). High rates of agoraphobic avoidance have been found in psychosis (Freeman et al., 2019a), social anxiety (Knapstad & Smith, 2021), panic disorder (Goodwin *et al*. 2005), and PTSD (Van Minnen & Hagenaars, 2010) suggesting that agoraphobic-type avoidance may be a final common pathway arising from many different types of fears, including concerns about negative judgement from self and others, panicking, and harm from others. Agoraphobic anxious avoidance of everyday situations is an important treatment target in its own right. We are trialling a new treatment for agoraphobic avoidance in patients with psychosis (Freeman et al., 2019b). This requires precise measurement to both identify the presence of a clinical problem accurately and to test treatment. This paper reports the development of an easy to use self-report measure of agoraphobic avoidance in psychosis based on the principles of behavioural avoidance tasks (BATs).

BATs are the gold standard for assessing anxious avoidance, providing an ecologically valid approach that is potentially objective. Participants are asked to approach a feared stimulus in a hierarchy of steps of increasing difficulty, stopping the progression when anxiety becomes unmanageable. This provides a measure of avoidance, based on the number of steps completed, and a measure of anxiety, based on subjective unit of distress (SUD) ratings obtained at each step. Versions of BATs have been used to assess anxious avoidance in phobias (Flatt & King, 2010; Ollendick, Allen, Benoit, & Cowart, 2011), social anxiety (Chorney et al., 2008; Compton et al., 2001; DiBartolo & Grills, 2006), Obsessive Compulsive Disorder (OCD) (Barrett & Healy-Farrell, 2003; Barrett, Healy, & March, 2003), PTSD (e.g. Saigh, 1989), and schizophrenia (Freeman et al., 2016). However, there is a clear drawback: they are often impractical. BATs are onerous to administer, lack standardisation, and do not lend themselves easily to psychometric evaluation. Few studies have assessed test–retest reliability (Hamilton & King, 1991). The result is that BATs are not used in clinical services and can be difficult to use in clinical research.

Our aim was to produce a self-report questionnaire of agoraphobic avoidance, The Oxford Agoraphobic Avoidance Scale (O-AS), based on the principles of BATs (i.e. assessing avoidance and anxiety in a series of steps for ecologically valid situations), that would be suitable for use in clinical services and research. We wanted a measure that was psychometrically robust, straightforward to complete, and focussed on everyday activities that are meaningful to patients. Using data from across the spectrum of severity, a combination of classical test theory (CTT) and item response theory (IRT) was used to develop the scale. CTT is based on the assumption that observed scores are determined by a person's true level of an underlying construct (e.g. agoraphobic anxious avoidance) and measurement error. This assumption underpins factor analytic techniques used to assess the dimensionality of a scale. In CTT the estimation of severity is based on a count of item endorsement within each dimension. In contrast, IRT examines the probabilistic relationship between the spectrum of the underlying construct and the ways in which individual items measure that construct. The differences between items are thus accounted for in the estimation of severity, and, as a result, IRT produces more precise estimates (Bortolotti, Tezza, de Andrade, Bornia, & de Sousa Júnior, 2013). Our objective was to use CCT and IRT to develop a precise measure of agoraphobic avoidance with a robust factor structure, assess the scale's item and test properties, and identify score ranges to aid use.

## Method

### Participants

We sought participants across the severity of agoraphobic avoidance. There were three groups: patients with psychosis who were experiencing agoraphobic avoidance severe enough for treatment, individuals from the general population meeting caseness for agoraphobia, and general population controls who did not meet caseness for agoraphobia. See Table 1 for demographic and clinical characteristics.

**Table 1.** Participant demographics and clinical characteristics

	Psychosis and agoraphobia (*n* = 194)	General population high agoraphobia (*n* = 427)	General population controls (*n* = 1094)
Age mean (s.d.)	37.3 (12.9)	42.2 (15.5)	45.9 (16.2)
Age range	16–71	18–77	18–86
	*n* (%)	*n* (%)	*n* (%)
Gender			
Male	143 (74)	100 (23.4)	303 (27.7)
Female	51 (26)	310 (72.6)	768 (70.2)
Nonbinary/prefer not to say	0	17 (4.0)	23 (2.2)
Ethnicity			
White	155 (79.9)	404 (94.6)	1437 (94.5)
Asian	2 (1)	7 (1.6)	25 (1.6)
Black	5 (3)	0	6 (0.4)
Mixed/multiple/other	18 (9.3)	11 (2.6)	41 (2.7)
Prefer not to say	0	5 (1.2)	11 (0.7)
Diagnosis			
Schizophrenia	84 (42)	–	–
Delusional disorder	2 (1)	–	–
Brief psychotic episode	7 (3.5)	–	–
Schizoaffective disorder	15 (7.5)	–	–
Other psychotic disorder	2 (1)	–	–
Unspecified psychotic disorder	56 (28)	–	–
Bipolar with psychotic features	3 (1.5)	–	–
Major depression with psychotic features	16 (8)	–	–
Current clinical service			
Early intervention team	72 (37.3)	–	–
Community mental health team	116 (60.1)	–	–
Inpatient service	5 (2.6)	–	–

The patients with psychosis were the first cohort of participants from gameChange, a randomised controlled trial of an automated virtual reality intervention to reduce agoraphobic avoidance in everyday situations (Freeman et al., 2019b). The main inclusion criteria for the gameChange trial were (1) a diagnosis of schizophrenia spectrum psychosis or affective disorder with psychotic symptoms and (2) self-reported difficulty going into everyday situations due to anxiety. This second criterion was established using a screening tool, the Brief Avoidance Scale (Freeman et al., 2019b). Participants had to report moderate to severe anxiety in three of the following situations: a café, pub, GP surgery, street, local shop, and bus; and want treatment to address this. In the gameChange sample, 93% scored above the clinical cut-off for agoraphobia on the Agoraphobia Mobility Inventory (AMI; score ⩾2.3).

Participants from the general population were recruited online using advertisements on social media. The inclusion criteria were: (i) being aged 18 years or older and (ii) resident in the UK. Caseness for agoraphobia was determined using the clinical cut-off for agoraphobia on the AMI (score ⩾2.3). To evaluate test–retest reliability, 264 participants from the general population (*n* = 94 with caseness for agoraphobia) repeated the questionnaire after 2 weeks. Ethical approval was obtained from the Medical Sciences Inter-Divisional Research Ethics Committee at the University of Oxford (R63059/RE001) for recruitment of the general population sample; and from the NHS South Central – Oxford B Research Ethics Committee (19/SC/0075) for recruitment of the psychosis sample.

### Assessments

#### Oxford Agoraphobic Avoidance Scale (O-AS) item pool

A pool of 40 items (see online Supplementary materials) was developed based on the principles of a BAT. Items reflected everyday activities, broken down into discrete steps of increasing difficulty (e.g. ‘Stand outside your home for 5 min’; ‘Walk down a quiet street’; ‘Walk down a busy street’). The items included activities in varied locations (e.g. around the home, outdoors, public transport, a GP surgery, shops, and cafés) that were either completed alone (e.g. ‘Sit in a café on your own for 10 min’) or with someone (e.g. ‘Sit in a café with someone you know for 10 min’). The content of items were generated through a discussion with a panel of clinical psychologists with expertise in treating agoraphobic anxious avoidance, review of individualised BATs used in previous studies, and through discussions with the gameChange Lived Experience Advisory Panel (LEAP), comprising 10 people with lived experience of psychosis and agoraphobic anxious avoidance. The final item pool was reviewed by both the LEAP and the panel of experts to ensure completeness, relevance, clarity, and readability. For each item, two rating scales are used. First, an avoidance rating is given for whether the participant thinks they could complete each task right now (‘Yes, I could do this now’ or ‘No, I would get too anxious’). Second, participants rate how anxious they would feel doing the task on a 0 (no distress) to 10 (extreme distress) scale. Higher avoidance and distress scores indicate higher levels of agoraphobia symptoms. The final scale can be seen in the Appendix.

#### Oxford behavioural avoidance task (O-BAT)

The O-BAT (Freeman et al., 2019b) is a real-world test involving a five-step hierarchy of situations that the participant finds difficult due to anxiety. The hierarchy is individualised for each participant and starts with a task that, while anxiety provoking, the individual thinks they would be able to do (green step). The next steps are tasks that the individual is uncertain whether they could complete due to anxiety (orange steps), and then, those that they could not complete due to anxiety (red steps). The O-BAT takes approximately 30 min to complete. The participant is asked to carry out each step, rate their anxiety for each step achieved, and stop when they are too anxious to continue. It provides an avoidance score based on the number of steps completed (0–5; with a lower score indicating higher avoidance) and a distress rating for each step completed from 0 (no distress) to 10 (extremely distressed).

#### Agoraphobia mobility inventory (AMI)

The AMI (Chambless, Caputo, Jasin, Gracely, & Williams, 1985) includes 26 items assessing avoidance of situations due to anxiety (i.e. agoraphobia). Items ask about avoidance of places (e.g. theatres, department stores, and museums), transport (e.g. airplanes, buses, cars), specific situations (e.g. being home alone, standing in queues), and spaces (e.g. enclosed spaces, high places, open spaces). Items are coded on a 1 (Never avoid) to 5 (Always avoid) scale. There is also an option to select ‘not applicable’; as a result, mean item scores are calculated. Higher mean scores indicate higher levels of agoraphobia. A score of >2.3 is used as an indication of agoraphobia caseness as recommended by IAPT NHS England (National Collaborating Centre for Mental Health, 2020). Cronbach' alpha for the AMI in the complete study sample (*n* = 1556) was 0.96.

#### Patient health questionnaire-9 (PHQ-9)

The PHQ-9 (Kroenke, Spitzer, & Williams, 2001) includes nine items assessing symptoms of depression over the past 2 weeks. Items are rated on a 0 (Not at all) to 3 (Nearly every day) scale. Higher scores indicate higher levels of depression. The Cronbach' alpha for the PHQ-9 in the complete study sample (*n* *=* 1464) was 0.93.

#### Generalised anxiety disorder-7 (GAD-7)

The GAD-7 (Spitzer, Kroenke, Williams, & Löwe, 2006) is a seven-item scale assessing symptoms of generalised anxiety over the past 2 weeks. Response options range from 0 (Not at all) to 3 (Nearly every day). Higher scores indicate higher levels of generalised anxiety. The Cronbach' alpha for the GAD-7 in the general population sample (*n* *=* 1412) was 0.94.

#### Revised-green paranoid thoughts scale (R-GPTS)

The R-GPTS (Freeman, Loe, et al., 2021) is a self-report measure assessing paranoid thinking over the past 2 weeks. The R-GPTS contains two separate scales assessing ideas of reference, RGPTS-R (8 items) and ideas of persecution, RGPTS-P (10 items). Items are rated on a 0 (Not at all) to 4 (Totally) scale, with higher scores indicating higher levels of paranoid thinking. Cronbach' alpha in the complete study sample (*n* *=* 1565) was 0.92 for ideas of reference and 0.95 for ideas of persecution.

#### Actigraphy

Actigraphy, which provides an objective measure of movement, was assessed using a Garmin Vivofit 4 watch. The Garmin watch uses macro movements to estimate the number of steps taken each day. Participants wore the watch for 5–7 days and a mean score of daily steps was calculated.

### Procedure

The new questionnaire item pool was completed by all three participant groups. To assess the concurrent validity of the final measure – the Oxford Agoraphobic Avoidance Scale (O-AS) – all participants completed the AMI, the R-GPTS scales, and the PHQ-9. Patients with psychosis also completed the O-BAT (after completing the self-report item pool) and provided actigraphy data. Participants in the general population also completed the GAD-7. Participants from the general population completed all measures online. Patients with psychosis completed the measures in person with the support of a research assistant.

### Statistical analysis

All analyses were conducted in R, version 3.6.1 (R Core Team, 2013). There were no missing data for the O-AS item pool since only participants with complete responses were included in the sample. For the additional measures, only responses from those who completed at least 80% of items on that measure were included. For questionnaires with less than 20% missing values, items were imputed using predictive mean matching in the ‘mice’ package (Buuren & Groothuis-Oudshoorn, 2011). Factor analysis was appropriate in both samples, as Bartlett's test of Sphericity was significant (psychosis sample: χ^2^ = 8784, df = 780, *p* < 0.001; general population sample: χ^2^ = 21830, df = 780, *p* < 0.001) and the Kaiser–Meyer–Olkin (KMO) test of sampling adequacy was excellent (psychosis KMO = 0.94; general population KMO = 0.96).

#### Development of the O-AS

To derive the O-AS from the item pool, exploratory factor analysis (EFA) was conducted using the ‘psych’ package (Revelle, 2020) with a combined sample of patients with psychosis and agoraphobia symptoms (*n* = 194) and participants from the general population meeting caseness for agoropohbia (*n* = 427). Items that were highly correlated with other items (r ⩾ 0.8) for either avoidance or distress scores were deleted prior to the EFA to avoid issues of multicollinearity. EFA was conducted with only the ordinal distress ratings (0–10) due to a greater variance in scores compared to the binary avoidance ratings. EFA was estimated using principal axis factoring to account for non-normality in the data (Costello & Osborne, 2005) and oblique rotation. Parallel analysis and inspection of scree plots were used to determine the number of factors to extract. Items were deleted if they were theoretically inconsistent or redundant (i.e. items not fitting with the theoretical understanding of the latent variable or items that are redundant as content is covered by another item), did not load onto any factor, or had cross-loadings above 0.30 on multiple factors.

Once a final set of items had been derived, confirmatory factor analysis (CFA) for both the avoidance and distress ratings was conducted to assess the model fit in (1) the combined agoraphobic group (n = 621) and (2) the complete sample (N = 1715). CFA was conducted in the ‘lavaan’ package (Rosseel, 2012) using the robust maximum likelihood (MLR) estimator for the ordinal distress scale and the robust weighted least-squares (WLSMV) estimator for the binary avoidance scale. A good model fit was determined using recommended thresholds of 0.95 (good) on the comparative Fit Index (CFI) and the Tucker–Lewis index (TLI), and <0.10 and <0.06 on the Root Mean Square Error of Approximation (RMSEA) and the Standardised Root Mean Square Residual (SRMR), respectively (Bentler & Bonett, 1980).

#### Evaluating psychometric properties

To examine the item and test properties of the O-AS, IRT analysis was conducted using the ‘mirt’ package (Chalmers, 2012) with the complete sample of patients with psychosis and all participants from the general population (N = 1715). The IRT analysis used a two-parameter graded response model (GRM) for the polytomous distress scale (Samejima, 1969), and a two-parameter logistic (2PL) model for the binary avoidance ratings (Baker & Kim, 2017). For both IRT analyses, outlier participants with atypical response patterns were excluded based on extreme person fit statistic scores (*z* < −3 or *z* > 3) (Felt, Castaneda, Tiemensma, & Depaoli, 2017). The IRT parameters are expressed as a function of theta (*θ*), representing the severity spectrum of the latent trait (i.e. agoraphobic avoidance). Higher θ values therefore reflect higher levels of agoraphobic avoidance.

#### Item properties

The IRT analyses produce discrimination and difficulty parameters for both O-AS ratings. The discrimination (a) parameter represents the ability of each item to discriminate levels of agoraphobic avoidance across the spectrum of severity. Higher discrimination values therefore indicate that the probability of item endorsement increases with only small shifts in severity. Discrimination parameters above 1 are considered highly discriminative whilst parameters below 0.5 are not acceptable (Baker & Kim, 2017). The difficulty parameters (b) represent the level of severity (i.e. *θ*) that an item typically measures. For the binary avoidance ratings, a single difficulty parameter (b1) represents the 50% probability of endorsing that item. For the polytomous distress ratings, 10 difficulty parameters (b1–b10) represent the 50% probability of responding at the boundary between the 11 response options (0–10). A higher b parameter suggests the response option reflects a higher (i.e. more severe) level of anxious avoidance.

Using the IRT parameters, differential item functioning (DIF) analysis was conducted to assess measurement invariance between the genders (male v. female), age groups (16–30 years; 31–50 years; 51+ years), and sample population (patients with psychosis v. general population) (Choi, Gibbons, & Crane, 2011). Significant item variance between the groups was determined by a beta (β) change >10% or a pseudo-R^2^ > 0.13 (Crane et al., 2007).

#### Test properties

The internal reliability of the O-AS scales was evaluated using the test information (TI) function from the IRT models. The TI represents the precision of the scale as a function of theta, thus showing its internal reliability at differing points on the severity spectrum (i.e. theta). The formula 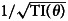 is used to convert TI at specific points of theta to a value that can be interpreted in line with Cronbach's alpha (O'Connor, 2018). To examine test–retest reliability in the 268 participants from the general population with repeat data, a two-way, mixed effects, absolute agreement intraclass correlation coefficient (ICC) was conducted.

Concurrent validity was examined using simple correlations between the O-AS scores and validated measures of anxious avoidance, anxiety, paranoia, and daytime activity (i.e. actigraphy data). An analysis of variance (ANOVA) was used to assess differences in O-AS scores between participant groups. The ecological validity of the O-AS was evaluated in the patient group by comparing scores with clinically assessed avoidance of real-world situations on the O-BAT. Items in the O-AS (e.g. ‘Order a drink on your own in a café’) were compared to equivalent steps on the O-BAT (e.g. ‘Order a drink in Costa on my own’), and the degree of concordance was calculated.

#### Determining score ranges

The expected score function from the IRT models and receiver operating characteristic (ROC) analyses were used to determine score ranges. The expected score function highlights the predicted score at each point of the severity spectrum, and its accuracy is determined by the fit of the IRT model to the data and the correlation between raw total scores and theta scores derived from the model. ROC analysis assessed the ability of the O-AS scales to distinguish the patients with agoraphobic avoidance requiring treatment in the context of psychosis (*n* *=* 194) and control participants from the general population (i.e. those without agoraphobia) (*n* *=* 1094). In ROC analysis, the area under the curve (AUC) represents a tests' discriminative ability, with values >0.70 considered fair, >0.80 good, and >0.90 excellent (Egan, 1975). Youden's *J* statistic is then used to determine a cut-off score with an optimal balance of sensitivity and specificity.

## Results

### Deriving the questionnaire

Using the combined agoraphobic avoidance sample (*n* *=* 621), 22 items were deleted prior to EFA due to high correlations with other items. During the EFA one item was deleted due to a low communality (<0.30), four items were deleted due to cross-loadings (>0.30), and five items were deleted for theoretical reasons. EFA on the distress score identified a single factor structure for the remaining eight items (including each of the six location domains) that explained 61% of the variance (see Table 2 for factor loadings). The correlation of *r* = 0.76 between the avoidance and distress scores suggested that although highly related, the two scales assess separate aspects of agoraphobic avoidance.

**Table 2.** Factor loadings for the O-AS distress scale from EFA derivation sample (*n* = 621) and Factor loadings for the O-AS distress and avoidance scales from CFA in full sample (*N* *=* 1715)

Item	Factor loadings		
	Distress (EFA)	Distress (CFA)	Avoidance (CFA)
1. Stand outside your home on your own for 5 min.	0.65	0.76	0.78
2. Walk down a quiet street on your own.	0.75	0.79	0.82
3. Walk down a busy street with someone you know.	0.79	0.85	0.84
4. Travel on your own on the bus for several stops.	0.79	0.88	0.88
5. Sit in the waiting room of your GP/health centre on your own for 5 min.	0.76	0.84	0.82
6. Purchase an item in a local shop, from a shop assistant.	0.82	0.88	0.88
7. Go to a shopping centre on your own for 15 min.	0.84	0.92	0.95
8. Sit in a café on your own for 10 min.	0.82	0.89	0.94

The eight-item unidimensional solution had a good model fit in the agoraphobia group for both the distress (χ^2^ = 108.7, df = 20, *p* < 0.001, CFI = 0.959, TLI = 0.943, RMSEA = 0.085, SRMR = 0.036) and the binary avoidance scores (χ^2^ = 60.7, df = 20, *p* < 0.001, CFI = 0.988, TLI = 0.983, RMSEA = 0.057, SRMR = 0.054). The structural validity of this unidimensional solution was further demonstrated in the complete participant group (*N* = 1715), where model fit was good for both the distress (χ^2^ = 180.3, df = 20, *p* < 0.001, CFI = 0.975, TLI = 0.965, RMSEA = 0.068, SRMR = 0.021) and the avoidance (χ^2^ = 64.6, df = 20, *p* < 0.001, CFI = 0.997, TLI = 0.995, RMSEA = 0.036, SRMR = 0.032) scores.

### Psychometric properties

IRT was conducted using the complete sample (*N* = 1715). For the distress scale, 11 participants were excluded due to high person fit statistics. A two-parameter GRM with the final sample (*n* = 1704) provided a good fit to the data (CFI = 0.99, TLI = 0.99, RMSEA = 0.070, SRMSR = 0.024). For the avoidance scale, no participants had high person fit statistics, and a 2PL model with all 1715 participants had an excellent model fit (CFI = 0.99, TLI = 0.99, RMSEA = 0.042, SRMSR = 0.032). All eight items had residual correlations below 0.20 on both scales, suggesting a lack of local dependence between items. The item parameters for both O-AS scales are shown in Table 3. Item probability trace lines and information functions are shown in the supplement.

**Table 3.** Discrimination (*a*) and difficulty (*b*) item parameters for O-AS avoidance and distress scales

Item	Avoidance		Distress										
	*a*	*b*1	*a*	*b*1	*b*2	*b*3	*b*4	*b*5	*b*6	*b*7	*b*8	*b*9	*b*10
1	1.24 (0.11)	1.99 (0.14)	1.60 (0.08)	0.40 (0.04)	0.68 (0.05)	0.99 (0.06)	1.28 (0.06)	1.52 (0.07)	2.02 (0.09)	2.30 (0.10)	2.66 (0.13)	3.15 (0.16)	3.49 (0.19)
2	2.56 (0.20)	1.33 (0.06)	2.59 (0.10)	−0.44 (0.04)	−0.10 (0.04)	0.23 (0.04)	0.51 (0.04)	0.75 (0.04)	1.04 (0.04)	1.26 (0.05)	1.51 (0.06)	1.92 (0.07)	2.29 (0.08)
3	5.43 (0.57)	1.03 (0.04)	4.45 (0.17)	−0.44 (0.03)	−0.11 (0.03)	0.16 (0.03)	0.34 (0.03)	0.53 (0.03)	0.71 (0.03)	0.88 (0.04)	1.07 (0.04)	1.33 (0.04)	1.65 (0.05)
4	3.59 (0.30)	1.14 (0.05)	4.14 (0.17)	−0.20 (0.03)	0.11 (0.03)	0.32 (0.03)	0.48 (0.03)	0.65 (0.03)	0.83 (0.04)	0.98 (0.04)	1.18 (0.04)	1.37 (0.05)	1.63 (0.05)
5	2.61 (0.23)	1.65 (0.07)	3.06 (0.12)	−0.26 (0.03)	0.02 (0.03)	0.32 (0.03)	0.52 (0.04)	0.74 (0.04)	0.99 (0.04)	1.15 (0.04)	1.37 (0.05)	1.65 (0.06)	2.01 (0.07)
6	3.18 (0.31)	1.73 (0.07)	3.93 (0.16)	0.03 (0.03)	0.33 (0.03)	0.57 (0.03)	0.77 (0.04)	0.97 (0.04)	1.16 (0.04)	1.34 (0.05)	1.50 (0.05)	1.83 (0.06)	2.15 (0.07)
7	5.36 (0.56)	0.99 (0.04)	5.48 (0.24)	−0.23 (0.03)	0.02 (0.03)	0.23 (0.03)	0.41 (0.03)	0.56 (0.03)	0.75 (0.03)	0.89 (0.04)	1.06 (0.04)	1.26 (0.04)	1.52 (0.05)
8	4.71 (0.44)	0.85 (0.04)	4.03 (0.15)	−0.51 (0.03)	−0.17 (0.03)	0.11 (0.03)	0.32 (0.03)	0.49 (0.03)	0.71 (0.04)	0.84 (0.04)	1.04 (0.04)	1.31 (0.04)	1.60 (0.05)

Standard errors in parentheses.

### Item properties

As shown in Table 3, all items for both the avoidance and the distress scales had high discrimination parameters, indicating small increases in agoraphobic avoidance lead to large increases in the probability that items would be endorsed. Across the two scales, the two most discriminating items were ‘Walk down a busy street with someone you know’ (avoidance: *a* = 5.43, s.e. = 0.57; distress: *a* = 4.45, s.e. = 0.17), and ‘Go to a shopping centre on your own for 15 mins’ (avoidance: *a* = 5.36, s.e. = 0.56; distress: *a* = 5.48, s.e. = 0.24).

The difficulty parameters show that endorsement of any of the binary avoidance items was indicative of elevated agoraphobic avoidance of at least 0.85 s.d.s above average. However, the parameters suggest that avoidance of the tasks ‘Stand outside your home on your own for 5 min’, ‘Purchase an item in a local shop, from a shop assistant’, and ‘Sit in the waiting room of your GP/health centre on your own for 5 min’ would indicate the most severe agoraphobic avoidance of 1.65–1.99 s.d.s above average.

The difficulty parameters also show that, for all items, the 0–10 distress scale assessed a broad range of agoraphobic avoidance from low to severe. High distress ratings (*b*8–*b*10, item responses 7+) on all items represented heightened agoraphobic avoidance at over 1 s.d. above average, while ratings of 9+ represented 1.5–3.5 standard deviations above average. Notably, higher difficulty parameters across all response options (Table 3) suggest that distress associated with the tasks ‘Stand outside your home on your own for 5 mins’, ‘Purchase an item in a local shop, from a shop assistant’, and ‘Walk down a quiet street on your own’ is especially indicative of severe agoraphobic avoidance.

For both avoidance and distress scales, there was no evidence of significant difference between men (*n* = 546) and women (*n* = 1128), participants in different age categories (16–30 years, *n* = 449; 31–50 years, *n* = 583; 51+ years, *n* = 664), or between the patients with psychosis (*n* = 194) and individuals from the general population (*n* = 1379).

#### Test reliability

**Fig. 1.**
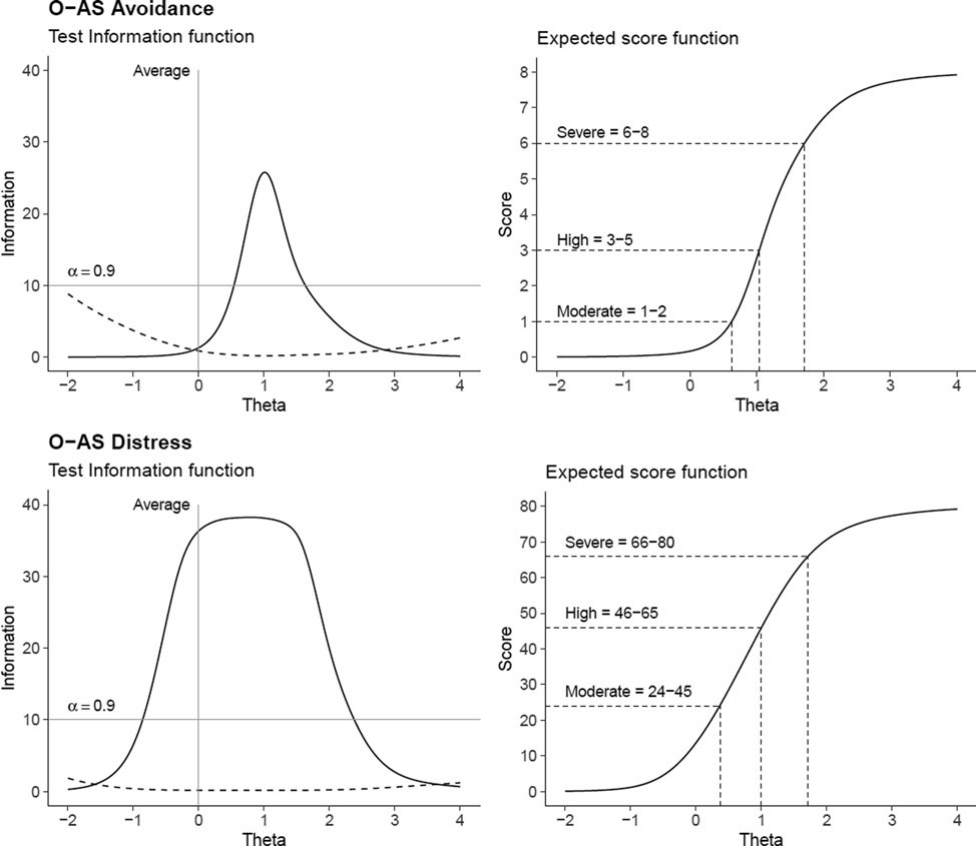
Test information

The TI functions shown in Fig. 1 represent the reliability of the O-AS as a function of agoraphobic avoidance severity (i.e. theta). These show that the avoidance score is highly reliable for the elevated levels of agoraphobic avoidance likely in clinical populations, with equivalent reliability of *α* > 0.90 (TI = 10) between 0.56 and 1.62 s.d. above average. However, the avoidance score had poorer reliability at the lower end of the severity spectrum with a limited ability to discriminate average levels (i.e. theta = 0). Conversely, the distress score demonstrated high reliability across a wider range of severity, with equivalent reliability of *α* > 0.90 between 0.86 s.d. below and 2.38 s.d. above average levels of agoraphobic avoidance. The maximum reliability of the avoidance score was *α* = 0.96 at 1.01 s.d. above average (TI = 25.8, s.e. = 0.20) and the maximum reliability of the distress score was *α* = 0.97 at 0.81 s.d. above average (TI = 38.2, s.e. = 0.16). Test–retest reliability after 2 weeks in the 264 participants from the general population was good for both avoidance [ICC = 0.83, 95% confidence interval (CI) 0.78–0.86] and distress (ICC = 0.91, 95% CI 0.89–0.93) scale.

### Score ranges

Both avoidance and distress scores demonstrated a high level of precision, with very high correlations between the summed total score and theta scores (avoidance: *r* = 0.96, distress: *r* = 0.95). Expected score functions derived from the IRT models are shown in Fig. 1. Mean scores and the proportions of individuals scoring above our recommended severity ranges in each sample group are shown in Table 4. ROC plots for both scores are shown in the supplement.

**Table 4.** Mean scores and proportions of participants scoring above each score range for the three participant groups

	General population controls (*n* = 1094)	General population high agoraphobia (*n* = 427)	Psychosis & agoraphobia (*n* = 194)
Avoidance			
Mean (s.d.)	0.31 (0.94)	2.17 (2.30)	3.34 (2.56)
	*n* (%)	*n* (%)	*n* (%)
0 Average	919 (84%)	149 (35%)	35 (18%)
1+ Moderate	175 (16%)	278 (65%)	159 (82%)
3+ High	42 (4%)	157 (37%)	111 (57%)
6+ Severe	7 (1%)	55 (13%)	49 (25%)
Distress			
Mean (s.d.)	9.38 (11.7)	37.0 (19.9)	52.3 (15.6)
	*n* (%)	*n* (%)	*n* (%)
⩽23 Average	965 (88%)	124 (29%)	8 (4%)
24+ Moderate	129 (12%)	303 (71%)	186 (96%)
46+ High	22 (2%)	149 (35%)	132 (68%)
66+ Severe	0 (0%)	33 (8%)	44 (23%)

#### Avoidance

As shown in Fig. 1, most people did not report avoiding any of the O-AS items, with an expected avoidance score of 0.17 (maximum score = 8) at the average level of trait agoraphobia. ROC analysis showed the avoidance score had good discriminatory power (AUC = 0.87, 95% CI 0.84–0.90). A score of 1/8 was the optimal cut-off for discriminating patients with agoraphobic avoidance in the context of psychosis (*n* = 194) from general population controls (*n* = 1094), with a sensitivity of 0.82 (95% CI 0.76–0.87) and a specificity of 0.84 (95% CI 0.82–0.86). Avoidance of at least one O-AS item represented agoraphobia levels of 0.62 s.d. above average and was used to define a moderately elevated score range. Avoidance of at least three items (⩾1 s.d.) defined a high score range and avoidance of more than six items (⩾1.7 s.d.) defined a severe range.

#### Distress

Unlike the avoidance score, most people are likely to report mild distress for some of the O-AS items, with an expected score of 13.6 (maximum score = 80) at average levels of trait agoraphobia. ROC analysis showed the distress rating has excellent discriminatory power (AUC = 0.97, 95% CI 0.97–0.98). A distress score of 24/80 was identified as the optimal cut-off for discriminating patients with agoraphobia and psychosis (*n* = 194) from general population controls (*n* = 1094), with a sensitivity of 0.96 (95% CI 0.93–0.98) and specificity of 0.88 (95% CI 0.86–0.90). A distress score of 24 represents 0.37 s.d. above average levels of trait agoraphobia and was used to define a moderately elevated score range. A distress score of at least 46 (⩾1 s.d. above average) defined a high range and a distress score of at least 66 (⩾1.70 s.d. above average) defined a severe range.

### Validity

Mean scores and correlations between the O-AS avoidance and distress scores and measures of agoraphobia, paranoid thoughts, depression, and generalised anxiety are shown in Table 5. The O-AS demonstrated good concurrent validity, with a strong correlation across the combined sample between both O-AS scales and an established measure of agoraphobia (i.e. AMI). Convergent validity with related psychological problems was also good. In the general population group, there was a moderate association between generalised anxiety and the O-AS avoidance scale and a strong association with the distress scales. Across the combined sample, there was a moderate association between O-AS scales and both paranoid thoughts and depression.

**Table 5.** Descriptive statistics and bivariate correlations between the O-AS and the other measures in the total sample, the general population (split into low and high agoraphobia subgroups), and patients with psychosis and agoraphobia

			Avoidance		Distress	
	*N*	Mean (s.d.)	*r*	*p*	*r*	*p*
Total sample	1715					
O-AS Avoidance	1715	1.12 (1.94)	–	/	/	/
O-AS Distress	1715	21.12 (21.78)	0.76	<0.001	/	/
Agoraphobia (AMI)	1556	2.11 (0.95)	0.71	<0.001	0.84	<0.001
Ideas of reference (R-GPTS)	1565	7.86 (8.07)	0.41	<0.001	0.55	<0.001
Ideas of persecution (R-GPTS)	1552	6.95 (9.88)	0.40	<0.001	0.52	<0.001
Depression (PHQ-9)	1464	11.98 (7.70)	0.44	<0.001	0.56	<0.001
General Population controls	1094					
O-AS Avoidance	1094	0.31 (0.94)	/	/	/	/
O-AS Distress	1094	9.38 (11.7)	0.59	<0.001	/	/
Agoraphobia (AMI)	1094	0.31 (0.94)	0.67	<0.001	0.81	<0.001
Ideas of reference (R-GPTS)	1094	9.38 (11.7)	0.42	<0.001	0.56	<0.001
Ideas of persecution (R-GPTS)	952	1.47 (0.38)	0.36	<0.001	0.47	<0.001
Generalised anxiety (GAD7)	985	14.4 (5.73)	0.46	<0.001	0.61	<0.001
Depression (PHQ-9)	975	4.81 (5.92)	0.47	<0.001	0.59	<0.001
General population high agoraphobia	427					
O-AS Avoidance	427	2.17 (2.30)	/	/	/	/
O-AS Distress	427	37.0 (19.9)	0.66	<0.001	/	/
AMI agoraphobia	427	3.05 (0.59)	0.61	<0.001	0.66	<0.001
Generalised anxiety	427	21.4 (5.21)	0.36	<0.001	0.44	<0.001
Ideas of reference	426	12.8 (8.54)	0.21	<0.001	0.30	<0.001
Ideas of persecution	427	11.2 (10.9)	0.17	<0.001	0.28	<0.001
Depression	403	17.4 (6.63)	0.40	<0.001	0.40	<0.001
Psychosis and agoraphobia	194					
O-AS Avoidance	194	3.34 (2.56)	/	/	/	/
O-AS Distress	194	52.3 (15.6)	0.65	<0.001	/	/
AMI agoraphobia	177	3.28 (0.70)	0.58	<0.001	0.56	<0.001
Ideas of reference	164	13.2 (8.81)	0.14	0.077	0.17	0.030
Ideas of persecution	164	16.1 (12.5)	0.17	0.026	0.24	0.002
Depression	173	15.0 (6.16)	0.19	0.011	0.32	<0.001
Actigraphy	87	4884.6 (3155.2)	−0.22	0.040	−0.29	0.006

Supporting the construct validity of the scale, there was a significant main effect of participant group on both O-AS avoidance (*F*_(2,1712)_ = 408.4, *p* < 0.001) and distress (*F*_(2,1712)_ = 1049, *p* < 0.001) scores. For both avoidance and distress scores, patients with agoraphobia in the context of psychosis scored significantly higher than the two general population groups (*p* < 0.001). Individuals in the general population meeting AMI caseness for agoraphobia then had significantly higher (*p* < 0.001) O-AS scores than the general population controls (see Table 5).

The relationship between the O-AS and real-world behaviour was supported by concordance with performance on the OBAT and correlations with actigraphy data. For the O-BAT, 955 steps from 191 participants were reviewed. Of these there were 191 steps that corresponded closely to an item on the O-AS. This included 39 green steps attempted by 39 different patients, 80 orange steps attempted by 70 different patients, and 72 red steps attempted by 62 different patients. There was good concordance between the O-AS and O-BAT (i.e. a patient rating they could or could not do the step on the O-AS and then completing or not completing that step during the BAT). The O-AS showed high concordance for green steps (82.1%) and red steps (76.4%). Concordance for orange steps, i.e. those designed to be something the patient is uncertain whether they can do, was moderate (53%). Actigraphy data in the psychosis sample showed a small but significant negative association with both the O-AS distress and avoidance scales, indicating higher scores were associated with less daily activity.

## Discussion

The Oxford Agoraphobic Avoidance Scale is a single factor, eight-item self-report questionnaire measuring agoraphobic avoidance. It presents everyday tasks of increasing difficulty, from standing outside the home to sitting in a café. Each O-AS item is rated on two separate response scales: avoidance (Yes/No) and distress (0–10). Although highly related, these two ratings function differently. Avoidance of any of the eight items is indicative of clinically elevated levels of agoraphobic avoidance and is highly reliable for assessing more severe presentations. The O-AS avoidance score will therefore function well as a clinical tool for patients with agoraphobic avoidance. In contrast, the distress ratings are able to detect a wide range of agoraphobic avoidance severity, from the mild anxiety that is common in the general population to the severe distress experienced by those using mental health services. The reliability of the distress score was very high across the severity spectrum, and therefore has good precision for use in both non-clinical and clinical populations. Convergent validity was shown with the gold standard of a BAT, and an objective measurement of movement. Test–retest reliability was also high for the questionnaire. Importantly, all items functioned similarly between gender, ages, and the different participant groups. The scale's strong psychometric properties, the ease of administration, and the frequency of agoraphobia across clinical conditions raise the likelihood of significant use.

The O-AS has a number of strengths compared to existing measures of agoraphobic anxious avoidance, the most commonly used of which is the AMI. The O-AS is briefer than the AMI, whilst still maintaining robust psychometric properties. Furthermore, O-AS items have been designed to reflect the everyday situations that patients consider important. This increases the utility of the O-AS as a meaningful outcome measure. In contrast, the AMI assesses a broad range of situations but many of these are not everyday (e.g. theatre, airplanes, high places). To the best of our knowledge, the O-AS is the first agoraphobia measure to be validated against a real-world behaviour avoidance task.

There are a number of limitations. The general population sample was recruited online and will not have been representative of the general population. Importantly, the high agoraphobia subsample from the general population was determined using scores from another self-report measure, the Agoraphobia Mobility Inventory, rather than from a structured diagnostic interview. However, we used the scale cut-off featured in the NHS Increasing Access to Psychological Therapy programme (<2.3; The National Collaborating Centre for Mental Health, 2018), which is more conservative than that recommended by the scale (<1.6; Chambless et al., 2011). A further limitation is that test–retest data were only collected for the general population sample. Sensitivity to clinically important change has also not yet been assessed. Our view is that agoraphobic anxious avoidance is a problem that significantly impairs the life of patients across a large number of mental health conditions – this precise assessment tool may help renew attention to the clinical issue.

## Supplementary material

The supplementary material for this article can be found at https://doi.org/10.1017/S0033291721002713

## Appendix

**Oxford – Agoraphobic Avoidance Scale (O-AS)**

Anxiety can make it difficult for people to go into everyday situations. This questionnaire is all about anxiety in everyday situations. We want to know whether there are situations that you do not go into because of anxiety. We also want to know how anxious you think you would feel if you were in each of these situations.

For each task below please tick whether or not you could do it at the moment (yes, I could do this now/no, I would get too anxious) and rate how anxious/distressed you think you would feel doing each task on a scale from 0 (No distress) to 10 (Extreme distress).

**Example:**
